# How funding structures for HIV/AIDS research shape outputs and utilization: a Swiss case study

**DOI:** 10.1186/1758-2652-14-S2-S7

**Published:** 2011-09-27

**Authors:** Kathrin Frey, Daniel Kübler

**Affiliations:** 1 Department of Political Science, University of Zurich Affolternstrasse 56, CH-8050, Zurich, Switzerland

## Abstract

**Background:**

Research policy in the field of HIV has changed substantially in recent decades in Switzerland. Until 2004, social science research on HIV/AIDS was funded by specialized funding agencies. After 2004, funding of such research was “normalized” and integrated into the Swiss National Science Foundation as the main funding agency for scientific research in Switzerland. This paper offers a longitudinal analysis of the relationship between the changing nature of funding structures on the one hand and the production and communication of policy-relevant scientific knowledge in the field of HIV on the other hand.

**Methods:**

The analysis relies on an inventory of all social sciences research projects on HIV in Switzerland that were funded between 1987 and 2010, including topics covered and disciplines involved, as well as financial data. In addition, in-depth interviews were conducted with 18 stakeholders.

**Results:**

The analysis highlights that the pre-2004 funding policy ensured good coverage of important social science research themes. Specific incentives and explicit promotion of social science research related to HIV gave rise to a multidisciplinary, integrative and health-oriented approach. The abolition of a specific funding policy in 2004 was paralleled by a drastic reduction in the number of social science research projects submitted for funding, and a decline of public money dedicated to such research. Although the public administration in charge of HIV policy still acknowledges the relevance of findings from social sciences for the development of prevention, treatment and care, HIV-related social science research does not flourish under current funding conditions.

**Conclusions:**

The Swiss experience sheds light on the difficulties of sustaining social science research and multidisciplinary approaches related to HIV without specialized funding agencies. Future funding policy might not necessarily require specialized agencies, but should better take into account research dynamics and motivations in the field of social sciences.

## Background

Since the beginning of the epidemic three decades ago, the conditions and possibilities of dealing with HIV infection, its treatment and strategies for prevention have changed substantially. In western countries, the feared health catastrophe did not occur. This is partly due to the success of primary prevention policies that focused on influencing individual behaviour through public campaigns and structural prevention. Since the very beginning of the epidemic, social science research on HIV/ AIDS has contributed strongly to the development of such policies [1,2]. Basic social science research provided important insights into dynamics of risk behaviours of stigmatized groups that were mainly affected (mostly, the focus was on homosexual men and intravenous drug users), as well those as in unconventional, “taboo” social relations (outside stable partnerships, sex workers). The research findings of social sciences were, and still are, utilized to develop and improve target group-specific prevention measures.

However, the legitimacy of social science research on HIV/AIDS - the necessity for major inputs by social sciences - has been disputed with the availability of effective antiretroviral therapies in the mid-1990s [1,3]. The role of clinical medicine grew considerably, and the focus on biomedical prevention technologies became stronger. Yet, these certainly positive advancements in the fight against HIV/AIDS have not created the technical “magic bullets” [1] to answer all the questions raised by the epidemic for western societies, and from that point, to allow us to ignore the social, economic, psychological and political dimensions. In spite of medical progress, HIV infection remains a major public health issue in western countries, with evidence of continuing or even increasing transmission of HIV in specific population groups, such as men who have sex with men or migrants from countries with high HIV prevalence. Many social and political scientists and practitioners emphasize that basic social science research is important for understanding the dynamics of the HIV epidemic and for developing effective policies [3,4].

In this context, the question of how basic social science research on HIV/AIDS can be successfully promoted becomes crucial. But the steering of science is not an easy task. Science policies, more generally, face the double-edged problem of how, on the one hand, to make utilizers of scientific results, such as public administrators, economists and members of civil society, acknowledge and respect academic freedom, and how, on the other hand, to get science interested in the problems of policy, industry and society. This dilemma has been aptly described as a principal-agent game [5].

In this wider context, the relationship between funding agencies and research actors is obviously pivotal [6] as the nature of these relationships is important for determining the responsiveness of research actors to external goal setting [7]. Against this background, this study focuses on the relationship between the funding policy and the production and transmission of scientific knowledge relevant for policy making in the field of HIV. How do structures, norms and interests within funding agencies shape the research output?

This article provides a longitudinal analysis of the Swiss research policy between 1987 and 2010. The analysis focuses on basic social science research in the field of HIV/AIDS. We use “social science research” as an umbrella term to include studies on HIV/AIDS by such disciplines as anthropology, cultural studies, economics, education, law, linguistics, media studies, political science, sociology and psychology. The term also includes studies often labelled as public health research or as social and behavioural sciences [1].

Switzerland has a concentrated HIV epidemic among men who have sex with men and in migrants coming from sub-Saharan African countries, which have a generalized HIV epidemic. Compared with other western European countries, the reported number of newly diagnosed HIV infections in Switzerland is rather high [4,8].

As in most other western countries, social science research on HIV/AIDS was specifically encouraged at the beginning of the epidemic in Switzerland. On the one hand, the national HIV prevention strategy has been, and still is, accompanied by fairly comprehensive surveillance and evaluation activities [4,9-11]. These activities are financed directly by the Swiss Federal Office of Public Health and have not been questioned so far.

On the other hand, specialized funding agencies were promoting basic social science research until 2004. After 2004, these activities were delegated to the main funding agency for scientific research in Switzerland, the Swiss National Science Foundation (SNSF). Thus, Switzerland provides an interesting case where research promotion policy has changed considerably in recent decades.

This article investigates the transformation of the funding mechanisms and analyzes the effects of the transformation on the funding of social science research on HIV/AIDS, on the thematic and disciplinary orientation of this research, and on the communication of research results to policy makers. Based on this analysis, we draw lessons for a funding policy in the age of an intense “re-medicalization” of the HIV problem.

## Methods

The empirical analysis is based on three data sources: (1) quantitative data of an inventory of all social science research projects conducted on HIV/AIDS by researchers based in Switzerland between 1987 and 2010; (2) financial data on the *public* funding of HIV/AIDS basic research, including social sciences, biomedical and clinical research between 1990 and 2010 (we were not able to collect financial data for 1987-1989); and (3) qualitative data from in-depth interviews with stakeholders [12]. The quantitative data on the social science research projects and the financial data were compiled from documents obtained from the research agencies in charge. Note that the inventory does not include research related to behavioural surveillance of HIV/AIDS (funded directly by the Federal Office of Public Health on a contract basis) [9,11].

The qualitative data were drawn from in-depth interviews with 18 stakeholders involved in social science research on HIV/AIDS since 1987. Interviewees were selected for their personal experience in funding, doing or using social science research on HIV/AIDS in the period under scrutiny. More precisely, they were researchers (four interviews), representatives of the various funding agencies (four interviews), users of research results in the public administration (nine interviews), and representatives of non-governmental organizations active in the field (one interview).

The interviewees were asked for their experience and assessment of past and current funding policies, and their experience and assessment of communication and utilization of social science research for the development and implementation of HIV/AIDS policy in Switzerland. The interviews were conducted between March 2005 and October 2006 by the first author of this paper. Interviews were tape recorded and transcribed, and analyzed using content analysis techniques [13].

## Results

### Funding structures in the field of social science research on HIV/AIDS

The research funding policy related to HIV/AIDS between 1987 and 2010 is divided into three phases, each characterized by specific funding structures. To analyze these structures, we use a typology proposed by Braun [14] that focuses on the funding agencies as the major actors distributing public funds to do research. Funding agencies are financed by the state in order to define and execute a large part of the science policy. They determine to some extent *what* will be investigated and by *whom* by distributing resources in a selective way among disciplines and investigators. In this way, they have a pivotal role in influencing the development of science [14].

Funding agencies are in an intermediate position between politics and science, and thus, they have to settle potentially conflicting considerations between policy relevance and scientific advancement. Braun [14] argues funding agencies’ positioning between politics and science shapes their structure, norms and interests, resulting in different ways of perceiving and responding to problems. He distinguishes between three ideal types of funding agencies:

1. *Political funding agencies* immediately serve the interests of a ministry and are obliged to respond to general, pressing and multifaceted problems raised by the parliament or the government. In order to find practical and applicable solutions, they are forced to create “hybrid communities” [14] involving upcoming and unconventional scientists from various disciplines. In this way, they foster multidisciplinary solutions.

2. *Strategic funding agencies* promote research in a particular problem area (e.g., health, environment) and have the mission of promoting all promising research paths in the respective domain, as well as responding to problems raised by the scientific community, laymen or politicians. These agencies promote both disciplinary research and the development of strategies to apply basic research results. In this way, they foster a fruitful combination of both reputed scientists and more unconventional investigators.

3. *Science-based funding agencies* support all disciplines of science and aim to foster the most promising scientific areas for knowledge advancement. This tends to result in a strong disciplinary orientation promoting main-stream research, and bears the risk of disjointed research efforts where disciplines tackle a problem totally independently from each other and incoherently.

Now let us analyze what types of research funding agencies were established in Switzerland between 1987 and 2010 in the field of HIV/AIDS.

The first phase, between 1987 and 1999, was shaped by the willingness of the government to take extraordinary measures to fight against a new and threatening infectious disease. It commissioned an AIDS research programme with a considerable budget. The Commission for the Control of AIDS Research (CCAR) was set up under the umbrella of the health ministry, i.e., the Swiss Federal Office of Public Health (SFOPH), and involved medical scientists and representatives of the ministry of education and science, as well as representatives of the Swiss National Science Foundation (SNSF).

In contrast with the funding policy of the SNSF, the proposal for the CCAR was that it had to not only fulfill the criteria of scientific quality, but also had to contribute to the fight against HIV/AIDS. Due to the fact that the commission applied the foundation’s international peer review procedure, it was considered to be quasi independent, and was well accepted by national and international scientists. Its research output, especially the Swiss Cohort Study, enjoyed a good reputation internationally [15,16].

In its early years, the commission received very few social science research proposals. This could be a result of the commission’s narrow focus and its domination by medical scientists. As one interviewee put it, “The CCAR realized that HIV/AIDS was related to many psychological and social problems that could not be solved by medical sciences. The researchers from medical science were overstrained and furthermore, they were faced with the dilemma that they considered social sciences not as true science. Therefore, it was decided to enlarge the commission and to put special emphasis on the fields of social sciences.”

Subsequently, the commission was enlarged by three social scientists in 1992 and started to pursue a more active promotion of submissions, including more detailed calls for proposal and the organization of conferences. Furthermore, it encouraged applicants with promising but not yet scientifically mature proposals to submit improved proposals for a second time. These measures contributed to the success of the CCAR in promoting not only biomedical and clinical research, but also social science research related to HIV/AIDS. The number of funded social science projects increased considerably in the mid-1990s. The CCAR succeeded in establishing an integrative health- and disease-oriented research concept [16,17].

**Figure 1:**
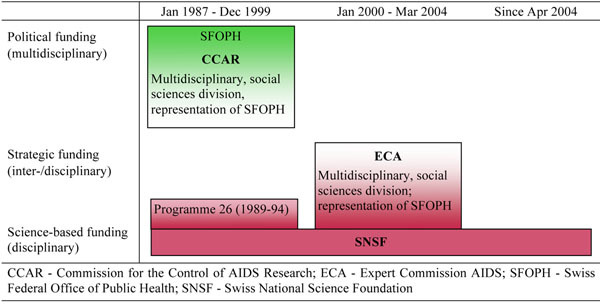
Funding structures of social science research on HIV/AIDS in Switzerland (1987 to present).

To sum up, the CCAR was a *political funding agency* that balanced its closeness to politics with the adoption of the peer review procedure of the SNSF (see Figure 1). Additional to the funding activities of the CCAR, the SNSF welcomed research proposals on HIV/AIDS from all disciplines, and in the early 1990s, conducted “National Research Programme 26”, authorized by the Federal Council. This programme dealt with the topic of “men, health and environment” including social science research projects related to HIV/AIDS [19].

In the second phase, the programme for AIDS research and its budget was transferred to the SNSF in January 2000. The interviewed experts considered this incorporation of the extraordinary structure of the CCAR into the main Swiss funding agency as a logical consequence after so-called “normalization”, i.e., when the advent of antiretroviral therapy in the mid-1990s transformed HIV from a deadly threat into a “normal” chronic disease [1,2,4,20-22]. From 2000 until 2003, a contract between the federal authority and the SNSF determined that the AIDS research programme had to be maintained within the SNSF. Therefore, the foundation appointed a special Expert Commission AIDS (ECA), with former members of the CCAR, including a representative of the SFOPH.

In this way, the continuation of a multidisciplinary approach and policy orientation was secured within an otherwise purely *science-based funding agency.* In other words, as a science-based funding agency, the SNSF adopted a *strategic funding scheme* related to HIV/AIDS. The representation of the SFOPH within the ECA was considered particularly important to pursue a strategic funding scheme that ensured policy relevance. As one former member of the commission put it in the interview, “The commission welcomed this [membership by the SFOPH representative], as it wanted to promote research that was useful to policy, to prevention, to those concerned by HIV. These questions were answered by the SFOPH representative.”

In the third and current phase, the special commission and the earmark budget for AIDS research within the SNSF was abolished in March 2004. The SFOPH and the former AIDS commission did not support this decision, but their influence on the SNSF is very restricted due to the fact that the SNSF is organized as an independent science-based funding agency. Of course, the SNSF still welcomes social science research proposals related to HIV/AIDS, but the proposals are evaluated within the standard review procedure organized along disciplines. Thus, research not fitting within the traditional boundaries of disciplines might be less successful. In the interviews conducted two years after the abolishment, researchers and policy makers clearly stated that scientific interests and traditional disciplinary orientations have gained weight at the expense of multidisciplinary, health- and disease-oriented AIDS research. The interviewees agreed that social science research is far more jeopardized by this policy change than biomedical and clinical research.

Figure 1 illustrates the changes of the funding structures by illustrating their position between politics and science.

### Disciplinary orientation of social science research on HIV/AIDS

**Figure 2:**
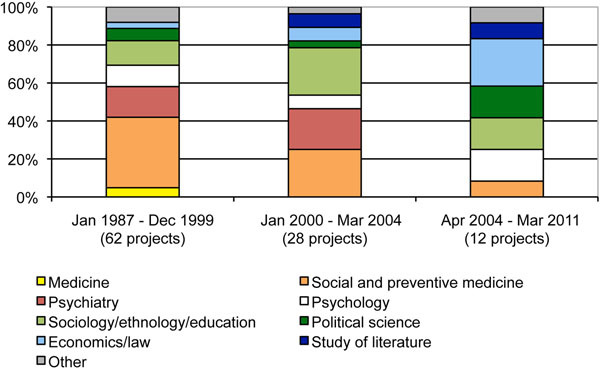
Disciplines of social science research projects on HIV/AIDS (1987-2011).

Between 1987 and 2010, these funding agencies financed a total of 102 projects in the field of basic social science research on HIV/AIDS. These projects cover a broad range of disciplines (Figure 2). All the funding agencies pursued the principle of competition, and research projects were conducted at various universities and research institutes located in German- and French-speaking regions of Switzerland. The competencies for social science research on HIV are therefore dispersed and dependent on individual researchers. Indeed, none of the research agencies in place followed a strategy to establish competence centres.

### Decline of funding for social science research on HIV/AIDS since 2004

**Figure 3:**
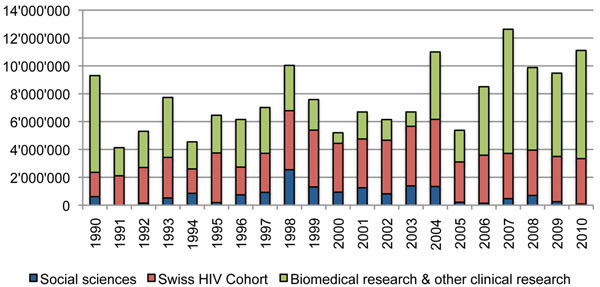
**Public funding for basic research on HIV/AIDS (in Swiss francs, 1990-2010).** For 1990 to 2003, only project funding of the CCAR and the ECA is included; the period of 2004 to 2010 comprises project funding and individual/career development funding of the SNSF. The data is comprehensive for the social sciences and the Swiss Cohort Study, but public funding for biomedical and other clinical research between 1990 and 2003 is not comprehensive because the separate funding of the SNSF is not included in this diagram. Sources: 1990-99: CCAR; 2000-2003: SNSF/ECA, 2004-2010: SNSF.

Figure 3 shows that public funding of social science research decreased drastically after the abolition of the strategic funding scheme in 2004, but the funds for HIV/ AIDS research in general were increased. Between 2000 and 2004, about 1.2 million Swiss francs per year were dedicated to the fields of social sciences. Thereafter, the funding for social science research on HIV/AIDS decreased drastically to 0.3 million Swiss francs per year. Only 3% of public money spent on HIV/AIDS basic research was dedicated to social sciences after 2004.

Thus, we witnessed not a decrease of public money being spent on HIV/AIDS research, but rather a marginalization of social sciences and a shift towards an even stronger concentration of public funding on biomedical and clinical research in the field of HIV/AIDS.

**Figure 4:**
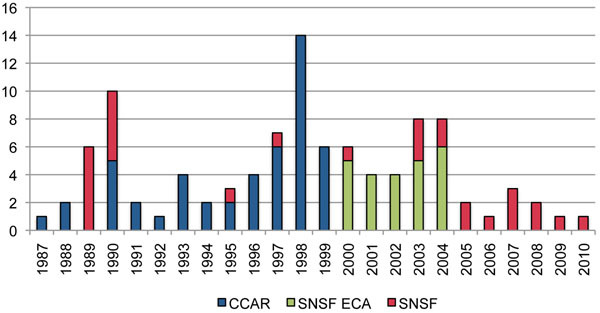
**Number of social science projects on HIV/AIDS, by funding agencies (1987-2010).** CCAR - Commission for the Control of AIDS Research; ECA - Expert Commission AIDS; SNSF - Swiss National Science Foundation. Sources: SNSF project data base, Swiss Foundation for Research in Social Sciences (FORS) Research Inventory; Swiss Federal Office of Public Health (SFOPH); CCAR.

Figure 4 shows that the number of social science research projects varied considerably between 1987 and 2010; however, after 2004, we can observe a decrease in the number of projects, parallel to the decline of funding. Since 2005, the SNSF has funded less than two social science research projects each year.

Table 1 shows that the decline in the number of funded social science projects on HIV/AIDS is strongly linked to a decrease in the number of research proposals submitted to the SNSF. Thus, the SNSF has not changed its approval rate; the decline in funded HIV/AIDS social science research projects is not caused by a stricter approval policy.

**Table 1:** Projects submitted to the SNSF in the field of social science research on HIV/AIDS

	2000	2001	2002	2003	2004	2005	2006	2007	2008	2009	2010	∅
Project proposals	10	7	7	14	13	4	2	4	7	2	2	6.5
Projects approved	6	4	4	8	8	2	1	3	2	1	1	3.6
Approval rate	60%	57%	57%	57%	61%	50%	50%	75%	28%	50%	50%	55%

Source: Swiss National Science Foundation (SNSF); including project funding and individual and career development funding.

The decline of submitted proposals as such is clearly a negative consequence of the abolition of the strategic funding scheme. The purely science-based funding structure in place since 2004 provides strong incentives for researchers to submit proposals within the traditional boundaries of their disciplines and oriented more towards mainstream research topics in the field of social sciences. Some social science researchers interviewed for the purpose of this study indicated that they or some of their colleagues have abandoned the focus on HIV/AIDS because they estimate their chances of success and reputation within their disciplines as rather marginal.

### Decline in communication between researchers and practitioners

Despite the popular idea to improve public policy by using evidence from research promoted since the late 1990s, research on the relation between evidence and policy acknowledges that this relation is complex and shaped by a myriad of intervening factors (e.g., lack of time, lack of analytical capacity within the public administration, ideological resistance, conflicting evidence) [23-26]. The point is that research findings do not automatically percolate into public policy.

In the present study, we concentrate on actors’ efforts to enhance the use of social science research, as well as on structural mechanisms linking researchers and users in the field of HIV [27,28]. On the one hand, researchers can facilitate research uptake by such efforts as making reports more readable and easier to understand, or focusing on variables that are amenable to interventions by users (“dissemination model”). On the other hand, users can invest more or less resources to collect, interpret and use research findings to improve policies (“demand pull model”). Linking mechanisms comprise formal and informal contact structures between researchers and users, such as joint committees or conferences (“interaction model”).

Our interview data indicate that actors’ efforts and the linking mechanisms in the field of HIV policy in Switzerland have changed considerably between 1987 and 2010. We can observe a shift from dissemination efforts by researchers to more intensive efforts by policy makers to stimulate basic social science research. Interview statements indicate that researchers’ commitment to considering the needs of the users and to contributing to the dissemination of their findings was stronger at the beginning of the HIV epidemic and decreased in the mid- and late 1990s as a concomitant of the normalization of HIV/AIDS. Thereafter, two strong linking mechanisms counteracted the fading commitment. Interviewees pointed out that, on the one hand, exchanges between researchers from various disciplines and research institutions, as well as between researchers and practitioners, continued to flourish thanks to a national conference organized yearly by the funding agency.

Interviewees mentioned that these conferences generated a discourse arena stimulating new research ideas and cooperation. As one of the interviewed researchers put it, “These conferences were an opportunity for building a community. They allowed (one) to get an overview of what was going on, to have in-depth discussions with one another, to start new cooperations. They often triggered ideas for a new project.”

On the other hand, the linkage between policy making and basic social science research was fostered by the representation of the SFOPH on the boards of the CCAR and the ECA between 1987 and 2004 (see Figure 1). Thereby, the SFOPH had the opportunity to share its assessment of the research proposals’ policy relevance during the evaluation process with other board members. Furthermore, this link guaranteed that the SFOPH was continuously informed about project submissions and approvals. After the abolition of the strategic funding scheme in 2004, these interaction mechanisms either ceased to exist or, in the case of the conference, faded away. After 2004, a national conference on social science and public health research in the field of HIV/AIDS in Switzerland took place twice, in 2005 and 2008, but this mechanism lost its power due to the great uncertainty caused by the abolition of the strategic funding scheme. The SFOPH tried to maintain the relation between researchers and practitioners by using and establishing more general communication channels. Furthermore, the SFOPH initiated and financed the present study, as well as other expertise [29], to shed light on the research policy and future opportunities to stimulate social science research in the field of HIV and sexual health in Switzerland. However, our data on the development of basic social science research in the field of HIV/AIDS indicate that in 2010, there is no sustained generation of findings in this research area.

## Discussion

In Switzerland, the structures for the promotion of publicly funded social science research related to HIV have substantially changed in the period under scrutiny (1987 to 2010). A science-based funding agency (the Swiss National Science Foundation) was in existence for the whole period. The political funding agency established in the early years of the epidemic (1987 to 1999) was replaced by a strategic funding scheme adopted by the SNSF and operational until early 2004; since then, the SNSF has solely allocated public funds for social science research on HIV.

The first change, from a political to a strategic funding scheme, was not paralleled by a significant change of HIV-related social science research, in terms of neither disciplines nor financial resources spent for this research. Both the political and the strategic funding schemes have contributed to the sustained production of social science knowledge on HIV-related issues, as well as to the continued transfer of this knowledge to policy makers. Social science research on HIV/AIDS in Switzerland has covered some important research issues discussed in the international literature [1,20,30], such as the vulnerability of affected population groups or discriminatory social conditions [4,12].

However, things changed after 2004, when the allocation of public funds for social science research related to HIV was limited to a purely science-based funding agency. While roughly five social science research projects in HIV/AIDS were executed per year in the first and second phase (i.e., when a political or a strategic funding scheme was present), the average dropped to roughly two projects per year after 2004. In parallel, funds for this type of research were reduced to a fourth of the average volume of previous years. However, this is not an effect of stricter approval policy, even though the approval rate dropped from a yearly average of 58.4% until 2004, to 50.5% after 2005.

Rather, it is an effect of a drastic reduction of the number of social science project proposals submitted to the funding agencies. While an average of 10.2 project proposals were submitted per year to the strategic funding agency before 2004, 3.5 social science projects proposals related to HIV were submitted to the science-based funding agency after 2004. Hence, the cause for reduced social science research activity in the field of HIV in Switzerland since 2004 lies in changed submission behaviour of researchers in the field.

Anticipating the disciplinary assessment standards of the science-based funding agency, researchers have tended to abandon the focus on HIV in formulating their proposals. This suggests that both the political and the strategic schemes, prior to 2004, have failed to establish HIV as a topic seen to be relevant by Swiss social scientists. This could be linked to the dispersed nature of projects funded at various universities across Switzerland. Some interviewees indeed pointed out that the establishment of research centres dedicated to social science research in field of HIV, or to sexual health more broadly, might have ensured sustainability of this type of research even after the major structural change in funding agencies in 2004. It is interesting to note that biomedical and clinical research on HIV did not face such problems of sustainability.

The reduced production of social science research after 2004 was paralleled by a shift in the practice of knowledge transfer to policy makers. We have seen that in the early years of the epidemic, transfer of scientific evidence to policy makers followed both a logic of dissemination (with researchers seeking to communicate research results to policy makers) and a logic of interaction (with policy makers suggesting policy-relevant issues and questions to researchers). This was certainly also linked to AIDS exceptionalism [2,22] after the onset of the epidemic, when policy makers and social science researchers were strongly motivated by the quest for ways and means to fight a new and threatening infectious disease.

Nevertheless, prior to 2004, such transfer of knowledge between social scientists and policy makers in the field of HIV/AIDS was fostered by a variety of instruments, including regular national conferences, as well as specific publication outlets, and also via the representation of policy makers on the boards of the funding commissions. With the progressive abolition or fading away of these various instruments since 2004, the knowledge transfer now follows essentially a demand pull logic: whether relevant scientific evidence produced by social scientists is found and used depends mainly on policy makers. The change of funding structures for social science research related to HIV in 2004 was paralleled by a reduction of actors’ investments into mechanisms that would enhance the communication and use of research findings in policy making. This situation clearly hampers the chances of social scientific evidence being used in HIV policy making compared with the situation prior to 2004.

## Conclusions

The Swiss experience sheds light on the difficulties of sustaining social science research related to HIV and its use in HIV policy making. The change in funding structures that occurred in 2004 reduced social scientists’ propensity to focus on HIV-related issues in their disciplines, and resulted in a decrease of projects and financial resources dedicated to such research, as well as a reduction of transfer activities between scientists and policy makers.

In the future, the changing dynamics of the HIV epidemic are likely to raise new issues and questions for policy making. Besides the contribution of biomedical and clinical sciences, the contribution of social sciences will also be crucial to the production of policy-relevant scientific evidence in this respect. Future funding policies for social science related to HIV might not necessarily require re-establishment of political or strategic funding schemes, but should better take into account disciplinary dynamics and foster researchers’ motivations to focus on these issues.

In Switzerland, this idea was acknowledged in a recent reformulation of the HIV prevention strategy: measures have been defined to foster coordination of social science research on HIV and other sexually transmitted infections, as well as the systematic use of scientific evidence in the development and implementation of prevention measures [31].

## Competing interests

Both authors declare that they have received funds for social science research (some of which focused on HIV) in the past and the present from the Swiss National Science Foundation.

## Authors’ contributions

DK was responsible for the study design. KF carried out the data collection, performed the analysis, and led the writing of the manuscript. DK contributed to final writing and editing. Both authors read and approved the final manuscript.
